# Adverse events, short- and long-term outcomes of extra corporeal liver therapy in the intensive care unit: 16 years experience with MARS® in a single center

**DOI:** 10.1186/s13054-022-04165-z

**Published:** 2022-09-19

**Authors:** Clément Monet, Audrey De Jong, Yassir Aarab, Lauranne Piron, Albert Prades, Julie Carr, Fouad Belafia, Gérald Chanques, Boris Guiu, Georges-Philippe Pageaux, Samir Jaber

**Affiliations:** 1 Department of Anesthesia and Intensive Care Unit, Regional University Hospital of Montpellier, St-Eloi Hospital, University of Montpellier, Montpellier Cedex 5, France; 2 PhyMedExp, INSERM U1046, CNRS UMR, University of Montpellier, 9214 Montpellier Cedex 5, France; 3 Department of Radiology, CHU Montpellier, Saint Eloi Teaching Hospital, University of Montpellier, Montpellier, France; 4 Liver and Transplantation Unit, University Hospital Montpellier, Montpellier, France

**Keywords:** MARS®, Liver dialysis, Liver failure, Pruritus, Critical care

## Abstract

**Background:**

Molecular Adsorbent Recirculating System (MARS®) is a non-biological artificial liver device. The benefit risk ratio between uncertain clinical effects and potential adverse events remains difficult to assess. We sought to describe adverse events related to MARS® therapy as well as biological and clinical effects.

**Methods:**

All intensive care unit (ICU) admissions to whom MARS® therapy was prescribed from March 2005 to August 2021 were consecutively and prospectively included. The main endpoint was the incidence of adverse events related to MARS® therapy. Secondary endpoints were the biological and clinical effects of MARS® therapy.

**Results:**

We reported 180 admissions treated with MARS® therapy. Among the 180 admissions, 56 (31.1%) were for acute-on-chronic liver failure, 32 (17.8%) for acute liver failure, 28 (15.5%) for post-surgery liver failure, 52 (28.9%) for pruritus and 12 (6.7%) for drug intoxication. At least one adverse event occurred in 95 (52.8%) admissions. Thrombocytopenia was the most frequent adverse event which was recorded in 55 admissions (30.6%). Overall, platelets count was 131 (± 95) × 10^9^/L before and 106 (± 72) × 10^9^/L after MARS® therapy (*p* < .001). After MARS® therapy, total bilirubin was significantly decreased in all groups (*p* < 0.05). Hepatic encephalopathy significantly improved in both the acute-on-chronic and in the acute liver failure group (*p* = 0.01). In the pruritus group, pruritus intensity score was significantly decreased after MARS® therapy (*p* < 0.01).

**Conclusion:**

In this large cohort of patients treated with MARS® therapy we report frequent adverse events. Thrombocytopenia was the most frequent adverse event. In all applications significant clinical and biological improvements were shown with MARS® therapy.

**Supplementary Information:**

The online version contains supplementary material available at 10.1186/s13054-022-04165-z.

## Introduction

Extra corporeal organ support devices have been developed during the last century to compensate for function loss in case of organ failure. Unlike renal replacement therapy, liver support therapy development has faced difficulties regarding the multiple and complex functions of the liver: protein synthesis and catabolism, detoxification, purification, biotransformation, glucose and lipid metabolism, excretion and immune modulation [1]. Existing methods consist of high volume plasma exchange and artificial liver devices based on albumin dialysis [2]. The Molecular Adsorbent Recirculating System (MARS®) is part of the last group. It is based on an albumin-enriched dialysis, allowing removal of albumin-bound toxins that accumulate during liver failure or, cases of cholestasis. Its purpose is limited to detoxification and purification [3]. Up to this day, there are no available guidelines on the use of MARS® therapy or any other liver support device. However, some potential clinical and/or biological improvements after liver support therapy have been reported in several types of liver failure: acute, acute-on-chronic and post-surgery liver failure [4–7]. Moreover, MARS® therapy has been used successfully to treat refractory pruritus secondary to cholestasis when medical treatment has failed [8]. Finally, MARS® therapy has been used to treat various intoxications with and without liver failure, particularly with drugs that are not removed by conventional hemodialysis, such as protein-bound drugs [9].

Experience and data on the use of MARS® therapy in the intensive care unit (ICU) are scarce and little is known on how to manage these procedures in ICU on a daily basis. We sought to describe our experience in MARS® therapy and report our practices. The main objective was to assess adverse events of MARS® therapy and the secondary objectives were to assess biological and clinical effects and applications of MARS® therapy. We hypothesized that MARS® therapy would be associated with frequent adverse events and positive biological and clinical effects.

## Materials and methods

### Patients and ethics

We report a retrospective, descriptive analysis of a prospective cohort of patients who underwent MARS® therapy in a single French center from March 2005 to August 2021. The Institutional Review Board of Montpellier University Hospital approved the study (2019_IRB-MTP_05-25). All admissions from March 2005 to September 2021 having been treated with MARS® therapy at any point during the ICU stay were included. These patients were divided into 5 groups depending on the indication for MARS® therapy: acute liver failure, acute-on-chronic liver failure, post-surgery liver failure (post hepatectomy and post transplantation), refractory pruritus and drug intoxication. These five indications were defined as usual: (1) acute liver failure was defined as the association of a rapidly evolving liver dysfunction with a drop of the prothrombin time and hepatic encephalopathy (any degree), provided that it affects a patient with a previously sane liver (illness < 26 weeks of duration) [10]; (2) acute-on-chronic liver failure combines an acute deterioration in liver function and organ failure(s) in patients suffering from chronic liver disease [11]; (3) post-surgery liver failure combines liver failure post hepatectomy or post transplantation as described previously [4, 12]; (4) refractory pruritus was defined as itching related to cholestasis without response to medical treatment [8]; (5) drug intoxication was defined as a comatose state secondary to drug absorption (patients with acute liver failure secondary to drug intoxication were included in the acute liver failure group provided that they met eligibility criteria).

### MARS® therapy applications

MARS® therapy was performed through a standard double lumen dialysis cannula placed in the femoral or jugular vein. The MARS® sessions were programmed for 8 h at a time on three consecutive days. We used the monitor MARS® 1 TC (Gambro, Baxter International, USA) coupled with the dialysis machine Prismaflex® (Baxter International, USA). Blood passed through a hollow-fiber, high-flux dialysis membrane (MARS Flux®; Gambro, Baxter International, USA). The blood flow was set at 180 ml/min. The albumin dialysate circuit consisted of 500 ml of 20% human albumin with a flow rate equivalent to the blood flow (180 ml/min). The albumin dialysate solution was regenerated by an anion-exchange column and an uncoated charcoal column (diaMARS® IE250 and diaMARS® AC250). Through a second membrane (diaFLUX®) the dialysis machine also helped purify the albumin dialysate solution by removing the soluble toxins. Anticoagulation was left to the clinician’s appreciation regarding coagulation tests and clinical data. Platelets transfusions were administered before the start of MARS® therapy if the platelets count was lower than 50 × 10^9^/L or at the clinician’s appreciation in case of bleeding risks. No systematic prophylactic antibiotic treatment was delivered during MARS® therapy. All patients received standard medical treatment of their condition as well as MARS® therapy.

### Endpoints

The main endpoint was the rate of adverse events. All adverse events that occurred during MARS® therapy sessions were recorded and were classified as follows: circuit-related, cannula-related, MARS®-associated thrombocytopenia and neurological events.

Adverse events were defined as follows: (1) culture proven infection of cannula: positive culture after removal of the cannula used for MARS® therapy; (2) cannula site bleeding: hemorrhage that required administering at least 1 unit of packed red cells or local treatment; (3) cannula dysfunction: requiring placement of a new cannula; (4) coagulation of the circuit or membrane: coagulation that led to premature ending of the session; (5) hypothermia: body temperature < 36 °C or complaint formulated by the patient; (6) arterial hypotension: hypotension that required vasopressors introduction or increase by 25% of the vasopressors dose or intravenous fluid therapy; (7) preemptive platelets transfusion: platelets transfusion performed before the start of MARS® therapy; (8) MARS®-associated thrombocytopenia: thrombocytopenia that required platelets transfusion (< 50 × 10^9^/L) from the day of the first session to the day after the last session in a patient without previous thrombocytopenia. In order to comply to the International System of Units (SI) we modified the platelets count unit to [platelets] × 10^9^/L.

Secondary endpoints were clinical and biological effects which were analyzed "after MARS® therapy" defined as after the end of all MARS® sessions during a same admission. Secondary endpoints were defined according to each group (Additional file 1: Table S1). In the acute liver failure, acute-on-chronic liver failure and post-surgery groups, biological and clinical effects were defined according to endpoints based on previous studies [5–7, 13–16]: (1) biological effects were based on total bilirubin, prothrombin time, albumin, alanine aminotransferase (ALT), aspartate aminotransferase (AST), gamma glutamyl transferase (GGT) and lactate levels; (2) clinical effects were based on Glasgow Coma Scale and hepatic encephalopathy which was assessed using West Haven criteria from 0 (no abnormality detected) to 4 (coma, unable to test mental state) [17]. In the refractory pruritus group, pruritus was evaluated at admission, before and after MARS® therapy with a numeric scale rating from 0 (no itch) to 10 (worst imaginable itch) [8, 18]. A decrease of intensity of pruritus by at least 50% on the numeric rating scale was defined as clinically relevant. Total bilirubin and bile acids were assessed in the refractory pruritus group. In the drug intoxication group, the effect of MARS® therapy was assessed by clinical endpoints: increase of Glasgow coma scale score as well as increase of the Richmond Agitation-Sedation Scale (RASS) score from deeply sedated (RASS of − 3 or less) to minimal sedation (RASS from − 2 to 0). MELD score was assessed in acute liver failure and acute-on-chronic liver failure groups. Survival at three and twelve months was recorded for all patients. Type of anticoagulation during MARS® therapy was also recorded in all patients. In case of clinical suspicion of heparin-induced thrombocytopenia type 2 during MARS® therapy, appropriate blood tests were performed according to the 4Ts clinical scoring system [19] (Additional file 1). If needed, we looked for anti-PF4/heparin antibodies.

### Statistical analysis

Continuous variables are presented as mean (± standard deviation) or as median with interquartile ranges [IQR]. Categorical variables are given in absolute numbers (percent proportion). Quantitative paired variables "Before" and "After" MARS® therapy were compared using a paired Student t-test or Wilcoxon signed-rank test when appropriate (failure of normality test). Qualitative paired variables were compared with a McNemar test. To explore the effect of time on the main outcomes, we performed a Mann–Kendall Trend Test. We also performed univariate and multivariate logistic regressions to assess the effect of time on adverse events and thrombocytopenia. We used the following adjustment variables: sex, age, and SOFA score. Statistical analyses were performed using the R software (R Foundation for Statistical Computing, Vienna, Austria). A *P* value < 0.05 was considered statistically significant.

## Results

### MARS® therapy applications

**Fig. 1:**
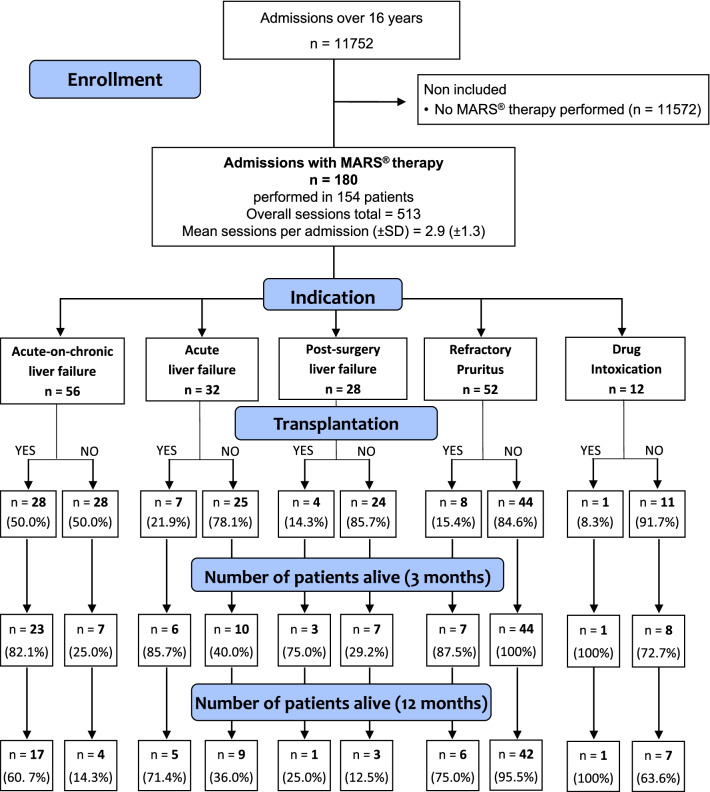
Flow chart and evolution of the 180 admissions included in the cohort

One hundred and eighty admissions of patients treated with MARS® therapy were included in the cohort. One hundred and fifty-four patients underwent 513 sessions (mean of 2.9 ± 1.3 sessions per admission). The mean duration of a session was 7 h 33 min (± 1 h 34 min). Among the 180 admissions, MARS® therapy was initiated in 56 (31.1%) admissions for acute-on-chronic liver failure, 32 (17.8%) admissions for acute liver failure, 28 (15.5%) admissions for post-surgery liver failure, 52 (28.9%) admissions for refractory pruritus and 12 (6.7%) admissions for drug intoxication (Fig. 1).

### Group description

Table 1 summarizes demographics and baseline characteristics of all patients.

**Table 1:** Patients' baseline characteristics

	Overall (*n* = 158)	Acute-on-chronic liver failure (*n* = 54)	Acute liver failure (*n* = 32)	Post-surgery liver failure (*n* = 25)	Refractory pruritus (*n* = 35)	Drug intoxication (*n* = 12)
*Demographics*						
Age (years)	54 [43–64]	56 [39–65]	58 [39–65]	58 [46–67]	48 [40–54]	51 [36–57]
Male gender (n)	99 (62.7)	41 (75.9)	18 (56.3)	19 (76.0)	13 (37.1)	8 (66.7)
BMI (kg/m^2^)	25 [21–28]	25 [21–29]	26 [23–29]	28 [22–29]	22 [19–24]	23 [20–25]
*Severity of illness*						
SOFA score	8 [4–10]	9 [7–10]	8 [6–11]	8 [10–12]	3 [1–4]	11 [8–15]
Respiratory	0 [0–1]	0 [0–1]	1 [0–1]	1 [0–3]	0 [0–0]	2 [1, 2]
Cardiovascular	0 [0–0]	0 [0–0]	0 [0–3]	3 [0–4]	0 [0–0]	2 [0–4]
Hepatic	3 [2–4]	4 [4]	3 [2–4]	4 [2–4]	2 [0–3]	0 [0–2]
Coagulation	1 [0–2]	2 [1, 2]	1 [0–2]	2 [1, 2]	0 [0–1]	1 [0–2]
Neurologic	0 [0–1]	0 [0–2]	0 [0–3]	0 [0–2]	0 [0–0]	4 [4]
Renal	1 [0–2]	2 [1–3]	1 [0–4]	1 [0–1]	0 [0–0]	2 [1, 2]
Catecholamines (n)	48 (30.4)	18 (33.3)	9 (28.1)	15 (60.0)	0 (0.0)	6 (50.0)
Child Pugh score	NA	11 [10–12]	NA	NA	NA	NA
MELD score	NA	34 [28–39]	28 [22–39]	NA	NA	NA

Baseline characteristics before MARS® therapy overall and in each group. Data are expressed as number (%) or median [interquartile range]

BMI, Body Mass Index; NA, not applicable; SOFA, Sequential Organ Failure Assessment; MELD, model for end-stage liver disease

#### Acute-on-chronic liver failure

Fifty-six admissions with MARS® therapy for acute-on-chronic liver failure were recorded for a total of 54 patients. MARS® therapy was performed for a median of 3 [2, 3] sessions per admission, time from admission to MARS® therapy was 4 [1–7] days. Causes of the underlying liver disease were: alcohol consumption (*n* = 24), chronic liver graft rejection (*n* = 6), hepatitis C alone (*n* = 6), secondary biliary cirrhosis (*n* = 5), combined alcohol consumption and hepatitis C (*n* = 3), primary sclerosing cholangitis (*n* = 3), non-alcoholic fatty liver disease (*n* = 2), primary biliary cirrhosis (*n* = 2), combined alcohol and non-alcoholic fatty liver disease (*n* = 1), drug-induced cirrhosis (*n* = 1) and autoimmune cirrhosis (*n* = 1). Factors triggering the acute liver decompensation were: sepsis or septic shock (*n* = 15), gastrointestinal bleeding (*n* = 8), acute hepatitis E (*n* = 4), acute alcoholic hepatitis (*n* = 3), iatrogenic (drugs, elective surgery) (*n* = 2), transjugular intrahepatic portosystemic shunt (*n* = 2), heart failure (*n* = 2), autoimmune hemolytic anemia (*n* = 1), hepatocellular carcinoma (*n* = 1), leptospirosis (*n* = 1) and portal vein thrombosis (*n* = 1). In 16 (28.6%) admissions, no triggering factor was found. Twenty-eight patients underwent a liver transplantation of which 82.1% survived at 3 months and 60.7% at 12 months (Fig. 1). Time from MARS® therapy to transplantation was 5 [3–13] days. Transplantation-free survival rate was 25.0% at 3 months and 14.3% at 12 months (Fig. 1).

#### Acute liver failure

Thirty-two admissions with MARS® therapy for acute liver failure were recorded for a total of thirty-two patients. MARS® therapy was performed for a median of 3 [2, 3] sessions per admission, time from admission to MARS® therapy was 4 [1–5] days. The etiologies of acute liver failure were: acetaminophen-induced (*n* = 11), drug-induced other than acetaminophen (antibiotics, voriconazole, amphetamines, others) (*n* = 4), acute viral hepatitis B (*n* = 4), ischemic hepatitis (*n* = 3), auto immune hepatitis (*n* = 3), acute alcoholic hepatitis (*n* = 2), acute viral hepatitis A (*n* = 1), heat stroke (*n* = 1), Langerhans cell histiocytosis (*n* = 1), AL amyloidosis (*n* = 1) and liver trauma (*n* = 1). Seven patients underwent a liver transplantation of which 85.7% survived at 3 months and 71.4% at 12 months (Fig. 1). Time from MARS® therapy to transplantation was 3 [1–9] days. Transplantation-free survival rate was 40.0% at 3 months and 36.0% at 12 months (Fig. 1).

#### Post-surgery liver failure

Twenty-eight admissions with MARS® therapy for liver failure after liver surgery were recorded for a total of 25 patients. MARS® therapy was performed for a median of 3 [2, 3] sessions per admission, time from admission to MARS® therapy was 14 [5, 9–15] days.

Six admissions were for hepatectomy further complicated with liver failure. The other 22 admissions were for liver transplantation further complicated with post transplantation liver failure, factors involved in the liver failure were: hemorrhagic shock (*n* = 11), graft dysfunction (*n* = 6), biliary peritonitis (*n* = 5), hepatic artery thrombosis (*n* = 2), cardiogenic shock (*n* = 1), infective endocarditis (*n* = 1) and pneumocystis pneumonia (*n* = 1). Four patients underwent a second liver transplantation of which 75.0% survived at 3 months and 25.0% at 12 months (Fig. 1). Time from MARS® therapy to retransplantation was 3 [3–5] days. Transplantation-free survival rate was 29.2% at 3 months and 12.5% at 12 months (Fig. 1).

#### Refractory pruritus

Fifty-two admissions were included with MARS® therapy for refractory pruritus for a total of 35 patients, the underlying diseases were: primary biliary cirrhosis (*n* = 19), chronic ischemic cholangitis (*n* = 11), acute cholestatic hepatitis (*n* = 3), cholestatic cirrhosis secondary to hepatitis C (*n* = 3), chronic rejection of liver graft (*n* = 3), alcoholic cirrhosis (*n* = 3), stenosis of the biliary ducts (*n* = 3), idiopathic cirrhosis with cholestasis (*n* = 2), primary sclerosing cholangitis (*n* = 2), autoimmune cholangitis (*n* = 1), secondary biliary cirrhosis (*n* = 1) and progressive familial intrahepatic cholestasis (*n* = 1). Three sessions of MARS® were performed per admission, except for 3 admissions that had two sessions and 2 admissions that had one session.

Eleven patients underwent a liver transplantation of which 87.5% survived at 3 months and 75.0% at 12 months (Fig. 1). Time from MARS® therapy to transplantation was 157 [29–248] days. Transplantation-free survival rate was 100% at 3 months and 95.5% at 12 months (Fig. 1).

#### Drug intoxication

Twelve admissions with MARS® therapy for drug intoxication without acute liver failure were recorded for a total of 12 patients. The median of sessions per admission was 3 [2, 3]. Reasons for initiating MARS therapy for drug intoxication were: delayed awakening after sedation with benzodiazepine (*n* = 6), iatrogenic status epilepticus in the context of antibiotic drug overdosage and renal failure (*n* = 3), self-induced benzodiazepine intoxication (*n* = 2) and overdosage of anti-epileptic drugs (*n* = 1). One patient was transplanted and alive at 12 months (Fig. 1). Time from MARS® therapy to transplantation was 2 days. Transplantation-free survival rate was 72.7% at 3 months and 63.6% at 12 months (Fig. 1).

### Main endpoint: adverse events

Adverse events are all reported in Table 2. Ninety-five admissions (52.8%) were associated with at least one adverse event. The main adverse event was MARS®-associated thrombocytopenia which was recorded in 55 (30.6%) admissions and in 65 (12.7%) sessions. Among patients with MARS®-associated thrombocytopenia, mean number of platelets concentrates transfusion was 2(**± **1.8). Preemptive platelets transfusion was recorded in 30 (16.7%) admissions. Overall, platelets count was 131(± 95) × 10^9^/L before and 106(± 72) × 10^9^/L after MARS® therapy (*p* < 0.001). One patient had an arterial misplacement of the cannula which required surgical intervention to remove, without any clinical repercussion. No case of type 2 heparin-induced thrombocytopenia was recorded. In this 16 years long cohort no effect of time on the main endpoints was observed (Additional file 1).

**Table 2:** Adverse events related to MARS® therapy in all patients

	Overall		Acute-on-chronic liver failure		Acute liver failure		Post-surgery liver failure		Refractory pruritus		Drug intoxication	
	Admissions	Sessions	Admissions	Sessions	Admissions	Sessions	Admissions	Sessions	Admissions	Sessions	Admissions	Sessions
	*n* = 180	*n* = 513	*n* = 56	*n* = 165	*n* = 32	*n* = 99	*n* = 28	*n* = 71	*n* = 52	*n* = 149	*n* = 12	*n* = 29
*Cannula-related complications*												
Culture proven infection of cannula	3(1.7)	3(0.6)	3(5.4)	3(1.8)	0(0)	0(0)	0(0)	0(0)	0(0)	0(0)	0(0)	0(0)
Cannula site bleeding	10(5.6)	10(1.9)	6(10.7)	6(3.6)	0(0)	0(0)	2(7.1)	2(2.8)	2(3.8)	2(1.3)	0(0)	0(0)
Cannula dysfunction	5(2.8)	5(1.0)	1(1.8)	1(0.6)	0(0)	0(0)	0(0)	0(0)	4(7.7)	4(2.7)	0(0)	0(0)
Cardiac arrhythmia related to cannula insertion	2(1.1)	2(0.4)	1(1.8)	1(0.6)	0(0)	0(0)	0(0)	0(0)	1(1.9)	1(0.7)	0(0)	0(0)
*Circuit-related complications*												
Coagulation of the circuit or membrane	36(20.0)	42(8.2)	13(23.2)	15(9.1)	8(25.0)	10(10.1)	4(14.3)	4(5.6)	8(15.4)	10(6.7)	3(25.0)	3(10.3)
Hypothermia	34(18.9)	34(6.6)	15(26.8)	15(9.1)	3(9.4)	3(3.0)	9(32.1)	9(12.7)	5(9.6)	5(3.3)	2(16.7)	2(6.9)
Arterial hypotension	21(11.7)	26(5.1)	7(12.5)	9(5.5)	3(9.4)	6(6.1)	5(17.9)	5(7.0)	4(7.7)	4(2.7)	2(16.7)	2(6.9)
*Thrombocytopenia associated to MARS®*												
Preemptive platelets transfusion	30(16.7)	36(7.0)	15(26.8)	19(11.5)	5(15.2)	6(6.1)	9(32.1)	10(14.1)	1(1.9)	1(0.7)	0(0)	0(0)
MARS®-associated thrombocytopenia	55(30.6)	65(12.7)	20(35.7)	25(15.2)	13(40.6)	14(14.1)	10(35.7)	12(16.9)	8(15.4)	9(6.0)	4(33.3)	5(17.2)
*Neurologic events*												
Epileptic seizure	3(1.7)	3(0.6)	1(1.8)	1(0.6)	1(3.1)	1(1.0)	1(3.6)	1(1.4)	0(0)	0(0)	0(0)	0(0)
CNS hemorrhage	1(0.6)	1(0.2)	0(0)	0(0)	1(1.0)	1(1.0)	0(0)	0(0)	0(0)	0(0)	0(0)	0(0)

Adverse events are presented by admission and by session for each group, *n*(%)

CNS, central nervous system

### Secondary endpoints: clinical and biological effects

Table 3 summarizes the main clinical and biological variables before and after MARS® therapy for each group.

**Table 3:** Clinical and biological variables before and after MARS® therapy in each group

	Acute-on-chronic liver failure			Acute liver failure			Post-surgery liver failure			Refractory pruritus			Drug intoxication		
	(*n* = 56)			(*n* = 32)			(*n* = 28)			(*n* = 52)			(*n* = 12)		
	Before MARS®	After MARS®	*p* value	Before MARS®	After MARS®	*p* value	Before MARS®	After MARS®	*p* value	Before MARS®	After MARS®	*p* value	Before MARS®	After MARS®	*p* value
Total bilirubin (µmol/L)	505 [311–642]	349 [228–403]	** < 0.01**	341 [188–520]	268 [109–353]	** < 0.01**	479 [337–551]	287 [176–347]	** < 0.01**	82 [27–185]	62 [23–142]	** < 0.01**	20 [11–112]	15 [11–61]	**0.01**
ALT (IU/L)	56 [34–117]	76 [43–175]	0.06	440 [121–1705]	250 [94–650]	** < 0.01**	153 [81–241]	100 [60–150]	**0.02**	71 [43–90]	50 [33–94]	0.07	96 [33–202]	88 [21–130]	0.67
AST (IU/L)	102 [63–173]	128 [62–209]	**0.03**	259 [124–696]	116 [82–234]	**0.02**	100 [61–174]	72 [50–101]	**0.04**	73 [55–120]	67 [49–110]	0.07	60 [36–102]	49 [24–148]	0.72
GGT (IU/L)	67 [35–205]	92 [33–232]	0.45	131 [41–314]	115 [56–307]	0.48	143 [81–260]	134 [93–297]	0.96	155 [89–421]	148 [68–385]	** < 0.01**	165 [69–253]	152 [48–242]	0.72
Prothrombin time (%)	42 [30–51]	47 [38–68]	** < 0.01**	39 [19–52]	60 [39–74]	** < 0.01**	61 [48–73]	63 [52–75]	0.56	90 [72–100]	82 [70–100]	0.64	78 [65–83]	78 [56–87]	0.76
Albumin (g/L)	33 [28–36]	31 [28–34]	0.20	30 [27–31]	31 [27–34]	0.12	30 [26–35]	30 [25–32]	0.34	33 [29–35]	33 [28–35]	0.11	28 [26–30]	29 [26–30]	0.96
Hemoglobin (g/dL)	9.0 [8.2–9.9]	9.4 [8.0–10.2]	0.83	10.7 [9.6–12.4]	9.5 [8.6–10.6]	** < 0.01**	9.2 [8.6–10.2]	9.2 [8.2–8.6]	0.14	11.0 [9.7–11.8]	9.6 [9.0–10.6]	** < 0.01**	9.2 [8.7–11.0]	9.3 [8.8–9.7]	0.13
Platelets count (× 10^9^/L)	80 [57–116]	72 [54–101]	**0.01**	97 [70–159]	94 [66–132]	0.19	92 [63–104]	77 [53–88]	0.15	167 [106–236]	116 [75–168]	** < 0.01**	116 [75–186]	163 [91–246]	0.97
Blood lactate concentration (mmol/L)	1.6 [1.2–2.1]	1.4 [1.0–1.8]	0.13	2.3 [1.7–2.8]	1.6 [1.2–2.5]	**0.01**	1.5 [1.2–2.2]	1.2 [1.3–1.8]	0.45	0.9 [0.7–1.4]	1.0 [0.9–1.4]	0.51	1.6 [1.2–2.3]	1.5 [1.0–1.9]	0.47
Glasgow coma scale score	14 [7–15]	15 [14, 15]	**0.01**	13 [4–15]	15 [11–15]	0.09	15 [9–15]	15 [9–15]	0.90	15 [15]	15 [15]	0.99	4 [3, 4]	15 [14, 15]	**0.01**
Encephalopathy (West Haven criteria)	2 [0–4]	1 [0–3]	**0.01**	2 [0–4]	0 [0–2]	**0.01**	1 [0–4]	1 [0–4]	0.99	0 [0–0]	0 [0–0]	0.99	NA	NA	

Continuous variables are presented as median with interquartile ranges [IQR]. Quantitative paired variables “Before” and “After” MARS® were compared using a paired Student t-test or Wilcoxon signed-rank test when appropriate (failure of normality test)

ALT, alanine aminotransferase; AST, aspartate aminotransferase; GGT, gamma glutamyl transferase

Bold indicates *p* value < 0.05 was considered statistically significant

#### Acute-on-chronic liver failure

Total bilirubin was significantly decreased from 505 µmol/L [311–642] to 349 µmol/L [228–403] (*p* < 0.01) and prothrombin time was significantly improved from 42% [30–51] to 47% [38–68] (*p* < 0.01) after MARS® therapy. Glasgow coma scale score and encephalopathy rate were significantly improved from 14 [7–15] to 15 [14, 15] (*p* = 0.01) and from 2 [0–4] to 1 [0–3] (*p* = 0.01) respectively after MARS® therapy.

#### Acute liver failure

Total bilirubin was significantly decreased from 341 µmol/L [188–520] to 268 µmol/L [109–353] (*p* < 0.01) and prothrombin time was significantly increased from 39% [19–52] to 60% [39–74] (*p* < 0.01) after MARS® therapy. Biological markers of liver necrosis were also significantly decreased after MARS® therapy, ALT levels from 440 [121–1705] IU/L to 250 [94–650] IU/L (*p* < 0.01), AST levels from 259 [124–696] IU/L to 116 [82–234] IU/L (*p* = 0.02) and blood lactate concentrations from 2.3 mmol/l [1.7–2.8] to 1.6 mmol/l [1.2–2.5] (*p* = 0.01).

Regarding clinical variables, hepatic encephalopathy score (West Haven criteria) was significantly improved from 2 [0–4] to 0 [0–2] (*p* = 0.01) after MARS® therapy.

#### Post-surgery liver failure

Total bilirubin was significantly decreased from 479 µmol/L [337–551] to 287 µmol/L [176–347] (*p* < 0.01). Biological markers of liver necrosis were also significantly decreased after MARS® therapy, ALT from 153 [81–241] IU/L to 100 [60–150] IU/L (*p* = 0.02), AST from 100 IU/L [61–174] to 72 IU/L [50–101] (*p* = 0.04).

#### Refractory pruritus

**Fig. 2:**
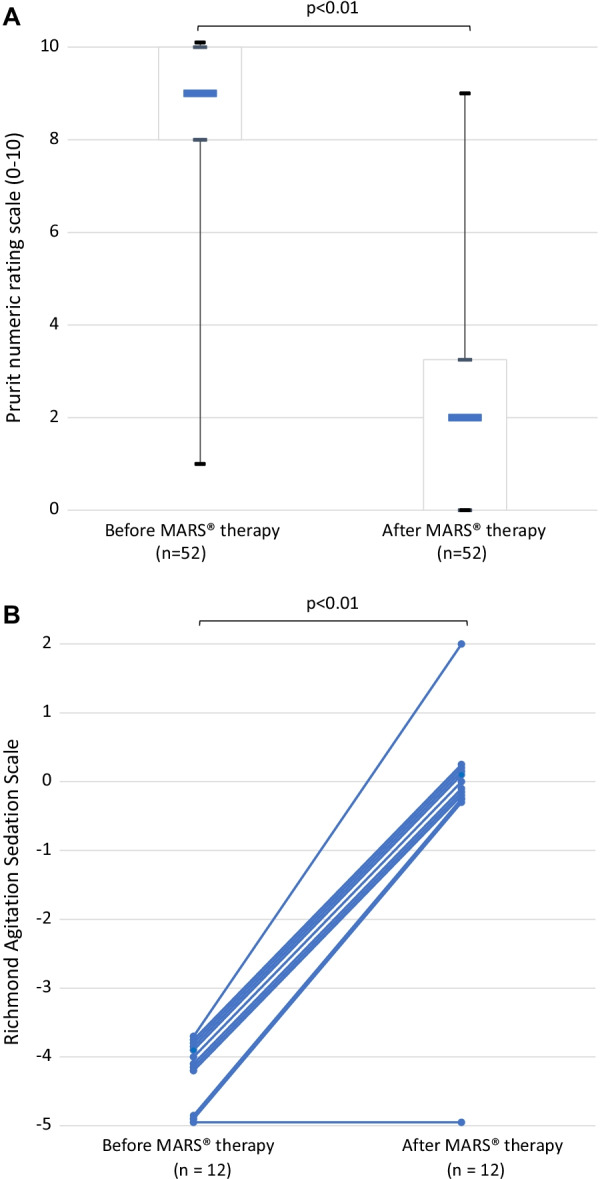
**A** Pruritus numeric rating scale before and after MARS® therapy in the refractory pruritus group. Numeric rating scale ranges from 0 (no itch) to 10 (worst imaginable itch). Patients perception of pruritus was decreased from 9 [8–10] to 2 [0–3] (*p* < 0.01) after MARS® therapy. Results are shown as median, first quartile, third quartile, minimum and maximum. **B** Richmond Agitation Sedation Scale (RASS) variation before and after MARS.® therapy in the drug intoxication group. RASS score improved in 11 out of 12 admissions (*p* < 0.01)

Pruritus was significantly decreased by at least 50% in 43 out of 52 admissions (*p* < 0.01) (Fig. 2A). After 3 sessions of MARS®, bile acids levels were significantly decreased from 95 [66–157] µmol/L to 48 [27–64] µmol/L (*p* < 0.01), total bilirubin was significantly decreased from 82 µmol/L [27–185] to 62 µmol/L [23–142] (*p* < 0.01) and serum GGT was significantly decreased from 155 IU/L [89–421] to 148 IU/L [68–385] (*p* < 0.01).

#### Drug intoxication

Initial RASS was − 4 or − 5 (deep sedation) in all admitted patients. In 11 out of 12 admissions, RASS was improved from deeply sedated (RASS of − 3 or less) to minimal sedation (RASS from − 2 to 0) after MARS® therapy (*p* < 0.01) (Fig. 2B). Glasgow coma scale score was also significantly improved after MARS® therapy from 4 [3, 4] to 15 [14, 15] (*p* = 0.01).

### Secondary endpoints: anticoagulation

The type of anticoagulation was: priming of the circuit only (2 L of saline solution with heparin, 2000 IU per liter) for 49.3% of the sessions, no anticoagulation for 16.8%, low molecular weight heparin (Enoxaparin) for 8.8%, anti-thrombin III infusion for 7.0% and heparin infusion at curative doses for 9.6%. Data regarding anticoagulation were not available for 8.6%. Anticoagulation in each group is detailed in Additional file 1: Table S2.

## Discussion

The main result of this study is that at least one adverse event occurred in 52.8% of admissions with MARS® therapy, the most frequent adverse event being MARS®-associated thrombocytopenia recorded in 55 (30.6%) admissions and in 65 (12.7%) sessions. This study is the largest single-center cohort (180 admissions in 154 patients) reporting exhaustive adverse events as well as both clinical and biological effects and 12-months survival in five highlighted applications of MARS® therapy: acute-on-chronic liver failure, acute liver failure, post-surgery liver failure, refractory pruritus and drug intoxication.

Hemodynamic tolerance was good with few episodes of arterial hypotension (11.7% of all admissions) which is consistent with previous studies results (7.7% [20], 16.7% [15]) although definitions may slightly differ and incidences were heterogeneous between studies. Hypothermia was an unexpected adverse event (18.9% of all admissions) although not surprising as it is a well-known adverse event of extra corporeal circuit of any type, the main explanation we found was the lack of systematic warmer activation on the dialysis device in the first years of MARS® therapy in our cohort. We report MARS®-associated thrombocytopenia as our main adverse event (12.7% of all sessions and 30.6% of all admissions). Three groups had a higher incidence of MARS®-associated thrombocytopenia: acute-on-chronic liver failure, acute liver failure and post-surgery liver failure groups (between 35.7% and 40.8% of admissions). However, these patients have multiple risks of thrombocytopenia other than MARS® therapy, the first and most obvious one being liver failure but also sepsis, surgery, acute gastrointestinal bleeding. We report platelets transfusion before the start of MARS® therapy (preemptive transfusion) in 16.7% of all admissions, this incidence has not been investigated elsewhere. It is worth noting that patients with platelets levels lower than 50 × 10^9^/L were excluded from most controlled trials on MARS® therapy, whereas they were included in this study after platelets transfusion. The refractory pruritus group has a particular relevance as it was mainly comprised of patients without major confounding factors since most of them were "out-patients" that were hospitalized in ICU for the sole stake of MARS® therapy. Therefore, the adverse events recorded in this group may be less influenced by exterior factors. Moreover, the benefit-risk ratio in these patients makes it particularly important to monitor adverse events as any adverse event in these patients without major organ failure would unbalance the benefit-risk ratio of MARS® therapy. In this particular group, preemptive platelets transfusion was recorded in only one admission and platelets count was significantly decreased from 167 [106–236] × 10^9^/L before to 116 [75–168] × 10^9^/L after MARS® therapy.

### MARS® therapy in acute-on-chronic liver failure

Total bilirubin and prothrombin time were significantly improved after a median of 3 sessions. These biological results are consistent with literature on MARS® therapy in acute-on-chronic liver failure as most studies have shown a decrease of total bilirubin [21–24] and an improved prothrombin time [21]. High levels of total bilirubin are associated with mortality in acute-on-chronic liver failure patients [25]. Clinically we observed a significant improvement of hepatic encephalopathy (using the West Haven criteria) and a significant improvement of Glasgow coma scale score. Most published studies show an improvement of hepatic encephalopathy (West Haven criteria) [20, 21, 26], interestingly we also observed an improvement of Glasgow coma scale score, which is a widely used scale to assess brain failure [27]. The 3-months transplantation-free survival was 25.0% in our cohort, which is similar to previously reported survival rates in acute-on-chronic liver failure patients [26, 28]. In the present study, all patients undergoing MARS® therapy were included as reflected by the median MELD score at admission of 34 (associated with a predicted mortality of 52.4% [29]) and the median SOFA score of 9 at admission (associated with a predicted mortality of 38.0% [30]). The inclusion of the most severe patients can be considered as a strength of the current study, as they have often been excluded from randomized controlled trials [26].

### MARS® therapy in acute liver failure

Liver injury biomarkers (total bilirubin, ALT, AST, prothrombin time and blood lactate concentrations) improved in the acute liver failure group after MARS® therapy. These results are similar to those found in previous controlled and uncontrolled studies [5–7, 13, 31–33]. Regarding clinical effects of MARS® therapy, hepatic encephalopathy was significantly improved, contrary to Glasgow coma scale score. These results are consistent with published studies that have shown improvement of hepatic encephalopathy [7, 13, 31]. One study also reported improvement of Glasgow coma scale [7]. There was a huge gap between transplanted and non-transplanted groups in terms of survival at 3 months (85.7% vs 40.0%). Survival in transplanted and non-transplanted patients was similar to those of published trials [7, 33] although transplantation-free survival after MARS® therapy differs across literature from 7%, 32% or 53% [7, 14, 34]. Indeed, prognosis of these patients is highly variable although liver transplantation has improved overall survival in acute liver failure patients [35]. In ALF patients, other extracorporeal therapies have been put forward such as high volume plasma exchange [36] or high dose continuous renal replacement therapy (CRRT) [37, 38]. CRRT could have a beneficial impact on hyperammonemia and consequently could lead to an improvement in transplantation free survival. It is another lead in the better management of ALF patients.

### MARS® therapy in post-surgery liver failure

In the post-surgery liver failure group, we showed significant decrease in total bilirubin, AST and ALT levels, which is consistent with biological effects found in published studies [12, 39–44]. Our post-surgery group is comprised of patients with either post-hepatectomy liver failure or post-transplantation liver failure, that is a similar cohort to the one reported by Kellersmann et al. [12] which found same results as ours regarding the decrease of total bilirubin. Among the 6 patients with post-hepatectomy liver failure, MARS® therapy was used as a "bridge to transplantation" for 2 patients who both underwent liver transplantation following MARS® therapy. Survival rate of post-hepatectomy liver failure patients seems to be poor in all available studies, mortality rate ranges from 80 to 100% in 3 published studies with 2, 4 and 5 patients respectively [12, 39, 40]. The other 22 patients of this group were treated for liver failure post liver transplantation [45]. Only a few series have been published about this application, with 1 to 15 patients treated with MARS® therapy [12, 39, 41–44]. Biological effects seem consistent in all studies, but survival is heterogeneous (from 0 to 100%), although these trials were not designed to assess such outcome and access to retransplantation is a major confounding factor in all these studies.

### MARS® therapy in refractory pruritus

We showed improvement of pruritus evaluated with a numeric rating scale before and after MARS® therapy which is coherent with published studies [8, 46–48]. To our knowledge it is the largest series of patients with refractory pruritus treated with MARS® therapy.

### MARS® therapy in drug intoxication

This specific application of MARS® therapy has not been well documented elsewhere, although MARS® therapy has been used to treat various intoxications without liver failure: calcium channel blocker, phenytoin and theophylline [49, 50]. Because of their particular pharmacokinetics (high protein fixation and liver bound biotransformation) benzodiazepines seem to fit the perfect profile of a molecule that could be purified by MARS® therapy, especially in case of chronic renal or liver insufficiency. No clinical data exists on benzodiazepine intoxication and MARS® therapy although there is animal evidence that protein-bound drugs such as midazolam and fentanyl are removed effectively from the plasma by MARS® therapy [51]. We showed clinical effects of MARS® therapy since Glasgow coma scale score and Richmond Agitation-Sedation scale (RASS) score were both improved. In selected patients in the ICU, especially those with renal and/or liver dysfunction, MARS® therapy could be a valuable option for reversal of benzodiazepine intoxication either self-induced or iatrogenic. In our study, patients treated for drug intoxication had fewer sessions compared to the other groups because efficacy or lack thereof was found rather quickly. It could potentially allow fewer days of mechanical ventilation and shorter ICU stays although our study was not designed to assess such outcome.

### MARS® therapy and anticoagulation

Anticoagulation was individualized to each patient and variations between patients and groups can be noted. Different factors can explain these variations. First, this cohort reflects 16 years of practice in our institution thus an evolution in knowledge and experience. Second, the wide variability within patients (from acute liver failure with multiple organ failure to pruritus without organ failure) leads to a variability in anticoagulation method and objectives. Although our study was not designed to assess this issue, no obvious relation was shown between anticoagulation and thrombosis of the membrane. Future considerations should include the questions of defining better criteria to decide which anticoagulation will provide the best advantage over risk ratio. Coagulation at the bed side (Quick test, thromboelastography, etc.) could be an option to better assess the need and the modality of anticoagulation during extra corporeal therapy. Regional citrate anticoagulation has also been evaluated on a limited number of patients and could be a safe anticoagulation option in the future [52, 53].

### Study limitations

Our study bears several limitations. The retrospective uncontrolled design of the study leads to potential biases that we tried to limit using a standardized prospective computer-based recording. The size of some subgroups was too small to obtain proper statistical power and results regarding the secondary endpoints should be considered as exploratory results. Moreover, heterogeneity of the post-surgery group is also a potential limitation for results interpretation as patients suffered from either brutal deterioration of the liver function following various intercurrent disease or rapidly worsening cholestasis in the same group. Our hematology laboratory did not always provide the International Normalized Ratio (INR) along the years of this study. Reasons for the INR not being always provided by the laboratory was its initial use only in patients taking vitamin K antagonists. In this study, prothrombin time is expressed as a percentage that stands for the time it takes for citrated plasma to clot in the presence of thromboplastin and calcium compared to a calibration line (or curve) established for a given calibrator and a given thromboplastin. All the prothrombin time results in the study come from the same laboratory therefore limiting the risk for errors due to differences in reagent. Finally, in our study, heparin was used in 9.4% and low molecular weight heparin in 8.9% of all admissions. Its use for MARS® therapy anticoagulation was limited in time (mean of 2.9 ± 1.3 sessions per admission and a mean time of 7 h 33 min (± 1 h 34 min) per session) and therefore estimated risk of heparin-induced thrombocytopenia (HIT) was low. No case of type 2 heparin-induced thrombocytopenia (HIT) was reported in this series. It is nonetheless possible that some MARS®-associated thrombocytopenia was linked to type 1 HIT.

Disseminated Intravascular Coagulation (DIC) is a challenging diagnosis that could also have been a confounding factor to MARS®-associated thrombocytopenia. Although, in patients with liver failure, the incidence of DIC is disputed and autopsy studies have shown little evidence for DIC [54].

### Future directions

A couple of issues and questions need better knowledge and investigations. First of all, judging criteria of efficacy should be better defined depending on subgroups. In the refractory pruritus group we could consider pruritus numeric scale as the main judging criteria of efficacy whereas transplantation-free survival may be the main criteria in the acute liver failure group and survival to transplantation or bridge to transplantation could be the objective of MARS® therapy in the acute-on-chronic liver failure group. In the end, short- and long-term mortality should always be monitored and could define efficacy of MARS® therapy. One strength of the present study is the assessment of long-term outcome (12 months’ survival). Better selection of patients who will benefit from MARS® should also be assessed. We also need better identification of patients who require anticoagulation and which type and better monitoring of antibiotics and sedative drugs during MARS®. Furthermore, optimal MARS® sessions duration remains to be determined, keeping in mind that during longer sessions adverse events may occur more frequently, although longer sessions could avoid a rebound effect that has been shown after MARS® sessions [39]. In a study by Camus et al. [32], sessions > 15 h were associated with improved liver function in acute liver failure patients. Cost utility of MARS® therapy also remains to be evaluated as it implies a certain investment. Kantola et al. [55] evaluated the cost-utility of MARS® therapy in 90 acute liver failure patients with the conclusion that MARS® therapy was less expensive and more effective than standard medical treatment in acute liver failure patients.

## Conclusion

In this large cohort of patients treated with MARS® therapy we report frequent adverse events, thrombocytopenia was the most frequent adverse event. Applications of MARS® therapy were: acute-on-chronic liver failure, acute liver failure, post-surgery liver failure, refractory pruritus and drug intoxication. In all applications significant clinical and biological improvements were shown with MARS® therapy. Large multicenter randomized controlled trials are needed to confirm these exploratory results.

## Supplementary Information

**Additional file 1:** 4 T’s score, Effect on time on main outcomes, Supplementary table 1 and 2.

## Data Availability

The datasets used and/or analyzed during the current study are available from the corresponding author on reasonable request.

## Abbreviations

ALT: Alanine aminotransferase
AST: Aspartate aminotransferase
BMI: Body Mass Index
GGT: Gamma glutamyl transferase
ICU: Intensive care unit
IQR: Interquartile range
MARS: Molecular adsorbent recirculating system
MELD: Model for end-stage liver disease
RASS: Richmond Agitation-Sedation Scale
SOFA: Sepsis-Related Organ Failure Assessment
