# Supplementary material for: Adverse events, short- and long-term outcomes of extra corporeal liver therapy in the intensive care unit: 16 years experience with MARS® in a single center
Source: Crit Care. 2022 Sep 19;26:282. doi: 10.1186/s13054-022-04165-z (PMC9484245; doi:10.1186/s13054-022-04165-z)
Supplement: Supplementary file 1 — Additional file 1: 4 T’s score, Effect on time on main outcomes, Supplementary table 1 and 2. [file 13054_2022_4165_MOESM1_ESM.docx]

**Supplementary file**

**Adverse events, short- and long-term outcomes of extra corporeal liver therapy in the intensive care unit:
 16 years experience with MARS^®^ in a single center**

Clément Monet ^(1)(2),^ Audrey De Jong ^(1)(2)^, Yassir Aarab ^(1)(2)^, Lauranne Piron ^(3)^, Albert Prades ^(1)^, Julie Carr ^(1)^, Fouad Belafia ^(1)^, Gérald Chanques ^(1)(2)^, Boris Guiu ^(3)^,

Georges-Philippe Pageaux ^(4)^, Samir Jaber ^(1)(2)^

## Supplementary Appendix

- 4Ts score
- Effect on time on main outcomes
  - Kendall Mann test
  - Univariate and multivariate logistic regression
- Supplementary table 1
- Supplementary table 2
- **4 T’s score**

| **4T's** | **2 points** | **1 point** | **0 point** |
| --- | --- | --- | --- |
| Thrombocytopenia | Platelet count fall >50% AND platelet nadir ≥20 | Platelet count fall 30–50% OR platelet nadir 10–19 | Platelet count fall <30% OR platelet nadir <10 |
| Timing of platelet count fall | Clear onset between days 5–10 or platelet fall ≤1 day (prior heparin exposure within 30 days) | Consistent with days 5–10 fall, but not clear (e.g. missing platelet counts); onset after day 10; or fall ≤1 day (prior heparin exposure 30–100 days ago) | Platelet count fall <4 days without recent exposure |
| Thrombosis or other sequelae | New thrombosis (confirmed); skin necrosis; acute systemic reaction post-IV unfractionated heparin (UFH) bolus | Progressive or recurrent thrombosis; Non-necrotizing (erythematous) skin lesions; Suspected thrombosis (not proven) | None |
| Other causes for thrombocytopenia | None apparent | Possible | Definite |

As described in the original reference : LO, G.K., JUHL, D., WARKENTIN, T.E., SIGOUIN, C.S., EICHLER, P. and GREINACHER, A. (2006), Evaluation of pretest clinical score (4 T's) for the diagnosis of heparin-induced thrombocytopenia in two clinical settings. Journal of Thrombosis and Haemostasis, 4: 759-765. https://doi.org/10.1111/j.1538-7836.2006.01787.x

- ***Effect on time on main outcomes***
- To explore the effect of time on the main outcomes, we performed a Mann-Kendall Trend Test for linear trend ; H0 (null hypothesis): there is no trend present in the data; HA (alternative hypothesis): A trend is present in the data. Three periods of time were defined: 2005-2010, 2011-2015 and 2016-2021. We tested the main outcomes for a trend in time: bilirubin before MARS^®^ therapy(p=0.53), bilirubin after MARS^®^ therapy(p=0.57), platelets count before(p=0.50) and platelets count after MARS^®^ therapy (p=0.57). No trend in time was found for these outcomes.
- We also performed an univariate logistic regression to analyse the relationship between time (dependent variable) and adverse events (independent variable) (p=0.20) and a multivariate logistic regression to account for the relationship between age (dependent variable), sex (dependent variable), SOFA (dependent variable), time (dependent variable) and adverse events (independent variable). After adjustment the relationship between time and adverse events remained non-significant (p=0.24). The same analysis was made to test for the relationship between time (dependent variable) and thrombocytopenia (independent variable) (univariate logistic regression, p=0.24). And we also performed a multivariate logistic regression to analyse the relationship between age (dependent variable), sex (dependent variable), SOFA (dependent variable), time (dependent variable) and thrombocytopenia (independent variable). Again, relationship between time and thromboctypenia remained non-significant after adjustment (p=0.67)

|  | **Acute-on-chronic liver failure** | | **Acute liver failure** | **Post-surgery liver failure** | | **Refractory pruritus** | | **Drug intoxication** |
| --- | --- | --- | --- | --- | --- | --- | --- | --- |
| **Adverse events** | |  | | |  | |  | |
|  | - Catheter related  - Device related  - Thrombocytopenia  - Epileptic seizure | | - Catheter related  - Device related  - Thrombocytopenia  - Epileptic seizure | - Catheter related  - Device related  - Thrombocytopenia  - Epileptic seizure | | - Catheter related  - Device related  - Thrombocytopenia  - Epileptic seizure | | - Catheter related  - Device related  - Thrombocytopenia  - Epileptic seizure |
| **Biological and clinical effects** | | | | | | | | |
| Biological | - Bilirubin  - Prothrombin time  - Albumin  - ALT  - AST  - GGT  - Lactate | | - Bilirubin  - Prothrombin time  - Albumin  - ALT  - AST  - GGT  - Lactate | - Bilirubin  - Prothrombin time  - Albumin  - ALT  - AST  - GGT  - Lactate | | - Bilirubin  - Bile acids | | ND |
| Clinical | - Glasgow coma scale  - Hepatic encephalopathy | | - Glasgow coma scale  - Hepatic encephalopathy | - Glasgow coma scale  - Hepatic encephalopathy | | - Pruritus numeric rating scale | | - Glasgow coma scale  - RASS |

***Supplementary Table 1. Description of MARS® experience: main and secondary endpoints.***

*Patients were classified in groups depending on the application of MARS®: acute-on-chronic liver failure, acute liver failure, post-surgery liver failure, refractory pruritus and drug intoxication. Adverse events as well as biological and clinical effects were evaluated in all groups, we defined endpoints to measure relevant effects specific to each application. ALT: alanine aminotransferase; AST: aspartate aminotransferase; GGT: gamma glutamyl transferase; ND: not done; RASS: Richmond Agitation-Sedation Scale.*

***Supplementary Table 2. Anticoagulation used during MARS^®^ therapy overall and in each group.***

|  | **Overall** | | | **Acute-on-chronic**  **liver failure** | | **Acute liver failure** | | **Post-surgery liver failure** | | | | **Refractory pruritus** | | | **Drug intoxication** | | |  |
| --- | --- | --- | --- | --- | --- | --- | --- | --- | --- | --- | --- | --- | --- | --- | --- | --- | --- | --- |
|  | Admissions | | Sessions | Admissions | Sessions | Admissions | Sessions | Admissions | | Sessions | | Admissions | | Sessions | Admissions | | Sessions |  |
|  | n=180 | | n=513 | n=56 | n=165 | n=32 | n=99 | n=28 | | n=71 | | n=52 | | n=149 | n=12 | | n=29 |  |
| Priming of the circuit | 84(46.7) | 253(49.3) | | 25(44.6) | 81(49.1) | 15(46.9) | 53(53.5) | 15(53.6) | 39(54.9) | | 21(40.4) | | 58(38.9) | | 8(66.7) | 22(75.9) | | |
| No anticoagulation | 35(19.4) | 86(16.8) | | 13(23.2) | 35(21.2) | 8(25.0) | 18(18.2) | 9(32.1) | 20(28.2) | | 3(5.7) | | 9(6.0) | | 2(16.7) | 4(13.8) | | |
| Low molecular weight heparin | 16(8.9) | 45(8.8) | | 0(0) | 0(0) | 0(0) | 0(0) | 0(0) | 0(0) | | 15(28.8) | | 43(28.9) | | 1(8.3) | 2(6.9) | | |
| Anti-thrombin III infusion | 11(6.1) | 36(7.0) | | 7(12.5) | 24(14.5) | 3(9.4) | 9(9.1) | 1(3.6) | 3(4.2) | | 0(0) | | 0(0) | | 0(0) | 0(0) | | |
| Heparin infusion | 17(9.4) | 49(9.6) | | 3(5.4) | 6(3.6) | 4(12.5) | 15(15.2) | 2(7.1) | 6(8.5) | | 7(13.5) | | 21(14.1) | | 1(8.3) | 1(3.4) | | |
| Data unavailable | 17(9.4) | 44(8.6) | | 8(14.3) | 19(11.5) | 2(6.3) | 4(4.0) | 1(3.6) | 3(4.2) | | 6(11.5) | | 18(12.1) | | 0(0.0) | 0(0.0) | | |

*Priming of the circuit: 2 liters of saline solution with 2000 IU heparin per liter; no anticoagulation: priming with saline solution; low molecular weight heparin: enoxaparin. Data are expressed in number of cases*
